# RACIPE: a computational tool for modeling gene regulatory circuits using randomization

**DOI:** 10.1186/s12918-018-0594-6

**Published:** 2018-06-19

**Authors:** Bin Huang, Dongya Jia, Jingchen Feng, Herbert Levine, José N. Onuchic, Mingyang Lu

**Affiliations:** 1 Center for Theoretical Biological Physics, Rice University, Houston, TX USA; 2 Program in Systems, Synthetic and Physical Biology, Rice University, Houston, TX USA; 3 Department of Bioengineering, Rice University, Houston, TX USA; 4 Department of Biosciences, Rice University, Houston, TX USA; 5 Department of Physics and Astronomy, Rice University, Houston, TX USA; 6 Department of Chemistry, Rice University, Houston, TX USA; 7 The Jackson Laboratory, Bar Harbor, ME USA

**Keywords:** Random circuit perturbation, RACIPE, Gene regulatory circuits, GRNs, Dynamical features, Statistical analysis

## Abstract

**Background:**

One of the major challenges in traditional mathematical modeling of gene regulatory circuits is the insufficient knowledge of kinetic parameters. These parameters are often inferred from existing experimental data and/or educated guesses, which can be time-consuming and error-prone, especially for large networks.

**Results:**

We present a user-friendly computational tool for the community to use our newly developed method named *ra*ndom *ci*rcuit *pe*rturbation (RACIPE), to explore the robust dynamical features of gene regulatory circuits without the requirement of detailed kinetic parameters. Taking the network topology as the only input, RACIPE generates an ensemble of circuit models with distinct randomized parameters and uniquely identifies robust dynamical properties by statistical analysis. Here, we discuss the implementation of the software and the statistical analysis methods of RACIPE-generated data to identify robust gene expression patterns and the functions of genes and regulatory links. Finally, we apply the tool on coupled toggle-switch circuits and a published circuit of B-lymphopoiesis.

**Conclusions:**

We expect our new computational tool to contribute to a more comprehensive and unbiased understanding of mechanisms underlying gene regulatory networks. RACIPE is a free open source software distributed under (Apache 2.0) license and can be downloaded from GitHub (https://github.com/simonhb1990/RACIPE-1.0).

**Electronic supplementary material:**

The online version of this article (10.1186/s12918-018-0594-6) contains supplementary material, which is available to authorized users.

## Background

Biological processes are orchestrated by complex gene regulatory networks (GRNs). To understand the operating principles of GRNs, mathematical modeling approaches [1, 2] have been widely used in various contexts, such as regulation of cell cycle [3], stem cell development [4], circadian rhythm [5], developmental pattern formation [6] and cell phenotypic switches in cancer [7–11]. To model the dynamics of GRNs, different computational algorithms have been developed [12], such as ordinary differential equations (ODEs)-based models [13], Boolean network models [14, 15], Bayesian network models [16], agent-based models [17], and reaction-diffusion models [18]. The ODEs-based models consider more regulatory details compared to Boolean or Bayesian network models and less computationally intensive than agent-based model and reaction-diffusion models, thus being a very attractive approach to simulate the operation of GRNs. GRN modeling has been integrated with methods to design and optimize the gene circuits in systems and synthetic biology [19–22].

It is believed that there is a core gene regulatory circuit underlying a GRN which functions as a decision-making module for one specific biological process [23, 24]. Identification of such core gene circuits can largely reduce the complexity of network modeling. Notably, the core gene regulatory circuit doesn’t function alone. Instead, its operation is usually regulated by other genes and signaling pathways (“peripheral factors”) that interact with the core circuit. Although the ODE-based and other modeling approach have been successfully applied to analyze the dynamics of the core gene circuits in certain scenarios, these approaches typically suffer from two issues. First, it is very difficult for traditional modeling approach to consider the effects of these “peripheral” factors due to their inherent complexity. Second, the modeling approaches are usually limited by insufficient knowledge of the kinetic parameters for many of the biological processes. In this case, the values of most parameters have to be inferred either by educated guess or fitting to the experimental results, which can be time-consuming and error-prone especially for large gene networks.

To deal with these issues, we previously established a new computational method, named *ra*ndom *ci*rcuit *pe*rturbation (RACIPE), to study the robust dynamical features of gene regulatory circuits without the requirement of detailed kinetic parameters [25]. RACIPE takes the topology of the core regulatory circuit as the *only* input and unbiasedly generates an ensemble of mathematical models, each of which is characterized by a unique set of kinetic parameters. For each mathematical model, it contains a set of chemical rate equations, which are subjected to non-linear dynamics analysis. From the ensemble of models, we can analyze the robust dynamical properties of the core circuit by statistical analysis. In RACIPE, the effects of the “peripheral factors” are modeled as random perturbations to the kinetic parameters.

Unlike the traditional ODEs-based modeling [26], RACIPE uses a self-consistent scheme to randomize all kinetic parameters for each mathematical model instead of relying on a particular set of parameters. Unlike other methods using randomization [27–30], RACIPE adopts a more carefully designed sampling strategy to randomize parameters across a wide range while satisfying the half-function rule, where each regulatory link has about 50% chance to be activated in the ensemble of RACIPE models. Also, unlike other methods to estimate parameters of ODEs from the experimental data [31, 32], RACIPE is designed to explore the robust features of the gene regulatory circuits in a much broader ranges of parameters even without the input of experimental data. Then, RACIPE-generated gene expression data and corresponding parameters can be analyzed by statistical learning methods, such as hierarchical clustering analysis (HCA) and principal component analysis (PCA), which provides a holistic view of the dynamical behaviors of the gene circuits. Notably, RACIPE integrates statistical learning methods with parameter perturbations, which makes it distinct from the traditional parameter sensitivity analysis [27, 30], parameter space estimation [31] and other randomization strategies [28, 29]. In addition, our previous work shows that robust gene expression patterns are conserved against large parameter perturbations due to the restraints from the circuit topology. Thus, we can interrogate the dynamical property of a gene circuit by randomization.

Without the need to know detailed kinetic parameters, RACIPE can 1) identify conserved dynamical features of a relatively large gene regulatory circuits across an ensemble of mathematical models; and 2) generate predictions on gain-of-function and loss-of-function mutations of each gene/regulatory link; and 3) discover novel strategies to perturb particular cell phenotypes. The application of RACIPE to a proposed core 22-gene regulatory circuit governing epithelial-to-mesenchymal transition (EMT) showed that RACIPE captures experimentally observed stable cell phenotypes, and the efficiency of various biomarkers in distinguishing different EMT phenotypes [25].

Here, we report a new computational tool that we developed to easily implement the random circuit perturbation method. In the following, we first discuss the implementation of RACIPE, including how the tool processes the input topology file of a gene network, estimates the range of parameters for randomization and solves stable steady states, etc. By applying RACIPE on a coupled toggle-switch circuit, we evaluate the computational cost of using RACIPE, detail the procedure on how to choose an appropriate number of RACIPE models and number of initial conditions for each RACIPE model to get converged simulation results for a gene circuit, and further illustrate how to do perturbation analysis using RACIPE. Lastly, we apply RACIPE on a published gene circuit governing B-lymphopoiesis [33] and show that RACIPE can capture multiple gene expression states during B cell development and the fold-change in expression of several key regulators between stages [34]. In summary, we expect RACIPE will be a valuable and user-friendly tool for the community to decipher the robust dynamical features of gene circuits in many applications.

## Implementation

**Fig. 1:**
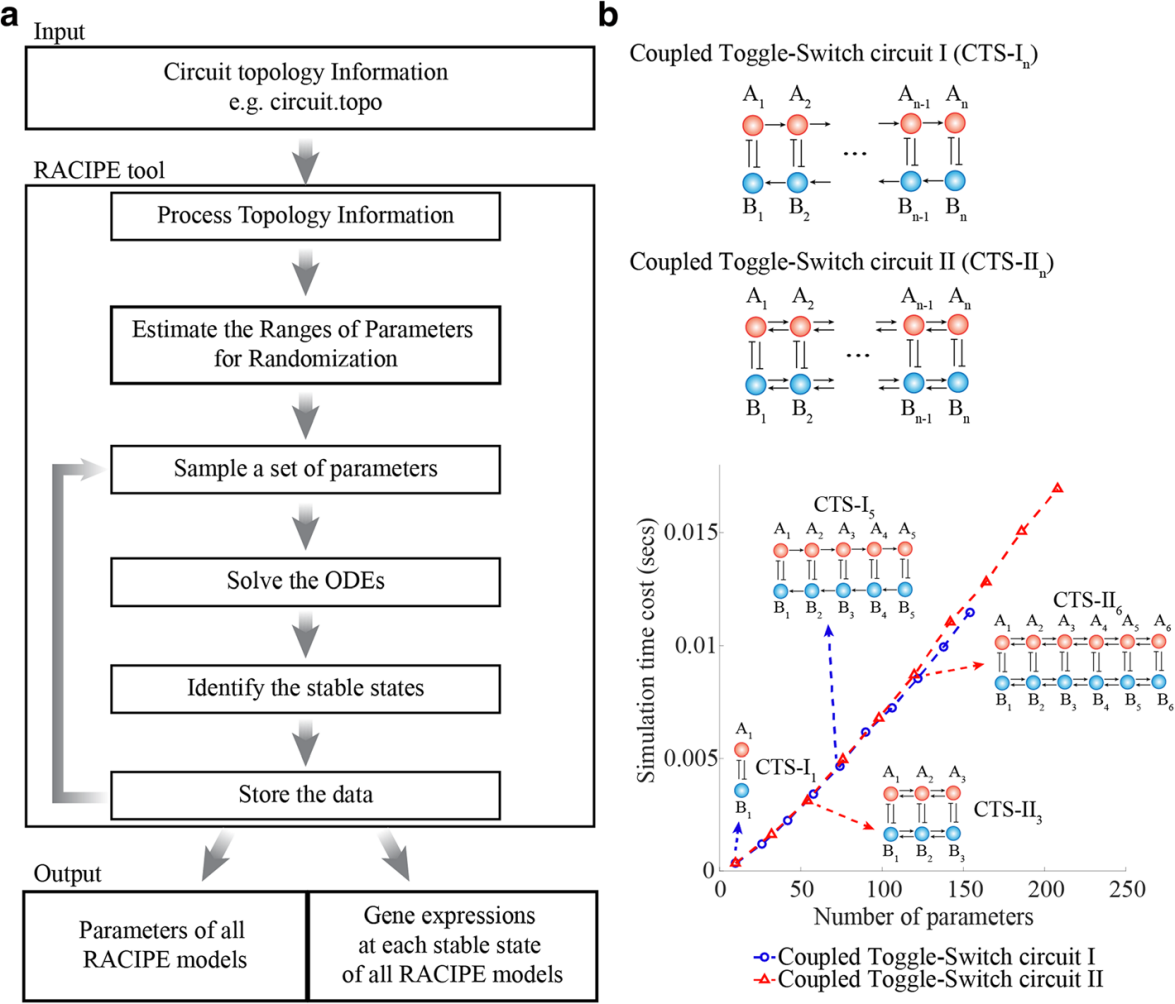
The computational tool of random circuit perturbation (**a**) Workflow of RACIPE. The only input for the tool is the circuit topology information. RACIPE automatically estimates the ranges of kinetic parameters for randomization and, from these ranges, randomly samples a particular set of parameters for a model. Then, it simulates the rate equations for this model to find all possible stable states. This procedure is repeated for many times to generate an ensemble of models. Finally, the tool outputs, from all the models, the kinetic parameters and the simulated gene expression of all stable states. **b** RACIPE is tested on two types of coupled toggle-switch (CTS) circuits (diagram illustrated in the top panel). The arrows represent transcriptional activation; the bar-headed arrows represent transcriptional inhibition. For both of the cases, the average time cost to simulate a RACIPE model (y-axis) is linearly proportional to the number of model parameters (x-axis)

RACIPE method is developed to identify the robust dynamical features of a biological gene circuit without the need of detailed circuit parameters [25]. RACIPE can generate and simulate an ensemble of models (Fig. 1a) and statistical analysis methods can be used to identify robust features of the circuit across all generated models. Here we report a newly developed tool based on the RACIPE method specifically for multi-stable gene regulatory circuits. With the input of the topology of a gene circuit, the tool automatically builds mathematical models for the circuit, randomizes the model parameters, and calculate the solutions of the stable steady states. These results can be used to uncover the robust features of the circuit, such as the stable steady-state gene expressions. The RACIPE tool currently can only calculate the solutions for the stable steady states but can be easily extended to study the temporal dynamics of a gene circuit. The main steps of the tool are elaborated below.

### Input data

The main input of RACIPE is the topology of a gene circuit, i.e. the gene names and the regulatory links connecting them. The current version can be applied to gene regulatory circuits with only transcription factors. We will expand its capacity to other regulation types in the future. In the input topology file (e.g., “circuit.topo”), each line specifies a regulatory link, which contains the name of the source gene, the name of the target gene, and the type of interactions (activation or inhibition). The list of gene nodes is not required, as it is automatically generated in RACIPE. Table 1 shows an example of the input topology file for a toggle-switch circuit, which has two mutually inhibiting genes A and B.

**Table 1:** Format of the input topology file (“circuit.topo”)

Source	Target	Type^#^
A	B	2
B	A	2

^#^“1” stands for activation, and “2” stands for inhibition

### Process circuit topology information

Based on the input circuit topology, RACIPE automatically builds mathematical models using ordinary differential equations (ODEs). For instance, the temporal dynamics of a toggle switch circuit can be modeled by the following ODEs:

$$ \dot{A}={G}_A{H}^S\left(B,{B}_A^0,{n}_{BA},{\lambda}_{BA}^{-}\right)-{k}_AA $$

1 $$ \dot{B}={G}_B{H}^S\left(A,{A}_B^0,{n}_{AB},{\lambda}_{AB}^{-}\right)-{k}_BB\kern0.5em $$

where *A* and *B* represent the protein levels of A and B encoded by genes A and B, respectively. *G*_*A*_and *G*_*B*_ are the maximum production rates (the production rate with all activators, but not any inhibitor, binding to the promoter region of the targeted gene). *k*_*A*_ and *k*_*B*_ are the innate degradation rates of the proteins A and B, respectively. The effects of the inhibitory regulation of gene A by B is formulated as a non-linear shifted Hill function [8] ${H}^S\left(B,{B}_A^0,{n}_{BA},{\lambda}_{BA}^{-}\right)$ defined as

2 $$ {H}^S\left(B,{B}_A^0,{n}_{BA},{\lambda}_{BA}^{-}\right),={\lambda}_{BA}^{-}+\left(1-{\lambda}_{BA}^{-}\right){H}^{-}\left(B,{B}_A^0,{n}_{BA}\right)\kern1em $$

where ${H}^{-}=1/\left(1+{\left(B/{B}_A^0\right)}^{n_{BA}}\right)$ is the inhibitory Hill function, ${B}_A^0$ is the threshold level, *n*_*BA*_ is the Hill coefficient and ${\lambda}_{BA}^{-}$ is the maximum fold change of the A level caused by the inhibitor B (${\lambda}_{BA}^{-}<1$). The inhibition of gene B by gene A can be modeled in a similar way. For gene circuits with excitatory links, the regulation of activation can also be modeled by the shifted Hill function, now with the fold change (*λ*) larger than 1.

When multiple regulators target a gene, the functional form of the rate equations depends on the nature of the multivalent regulation. Currently, we adopt a common scheme where we assume that these regulatory interactions are independent. Thus, the overall production rate is written as the product of the innate production rate of the target gene and the shifted Hill functions for all the regulatory links. We will consider other cases, such as competitive regulation, in a later version.

### Estimate the ranges of parameters for randomization

Next, RACIPE estimates, for each parameter, the range of values for randomization. Most of the parameter ranges, such as the ones of production and degradation rates, are preset (see Additional file 1: SI 1.1), while the ranges of the threshold values in the shift Hill functions are estimated numerically to satisfy the “half-functional” rule. The “half-functional” rule ensures that each link in the circuit has roughly 50% chance to be functional across all the models [25]. All the parameter ranges are generated and stored in a parameter file (“circuit.prs”).

### Solve and identify the stable steady states

To generate a model, RACIPE randomizes each parameter independently within the pre-calculated range. For each model with a particular set of parameters, RACIPE numerically simulates the dynamics of the model (see Additional file 1: SI 1.2). To identify all possible stable steady states of each model, RACIPE repeats the simulations for multiple times with different initial conditions, randomly chosen from a log-uniform distribution ranging from the minimum possible level to the maximum possible level. The stable steady states can be obtained in RACIPE by simulating the dynamics using the Euler method or the Runge-Kutta method. From the steady state solutions of all the realizations, we identify distinct stable states, defined as those whose Euclidean distances of the levels among them are all larger than a small threshold (see Additional file 1: SI 1.3). The above procedure is repeated for all the models. Together, we obtain a large set of gene expression data and model parameters for statistical analysis. In the implementation, RACIPE randomly generates a number mathematical of models, each of which is subject to simulations from a number of initial conditions. We will discuss how to appropriately choose the number of RACIPE models and the number of initial conditions for each RACIPE model in the Results section.

### Output data

Lastly, the model parameters and the steady state gene expressions of all RACIPE models are stored separately. The parameters for each RACIPE model are stored in “circuit_parameter.dat”, where each row corresponds to one RACIPE model, and each column shows the value of a parameter. The parameters follow the same order in the “circuit.prs” file. Depending on the number of stable states of a RACIPE model, its gene expressions are stored in the “circuit_solution_i.dat”, where i is the number of stable states. In the “circuit_solution_i.dat”, each row shows the gene expression vectors of all the stable steady states from a RACIPE model. These data are subject to further statistical analysis.

### Options

RACIPE allows adjusting simulation parameters by directly specifying them in the command line or in the “circuit.cfg” file (see the README file for detailed instructions). RACIPE allows the user to choose different ODE solvers (the first-order Euler or the Runge-Kutta method) and to export any RACIPE model into the SBML format [35, 36]. Moreover, RACIPE also has options to perform simulations of perturbations, such as gene knockout, gene overexpression and knockdown, and removal of a regulatory link*.* Unlike conventional approach, RACIPE applies perturbations (see Additional file 1: SI 1.4) to the entire ensemble of models to capture the conserved behaviors of the treatment.

## Results

### Time cost of simulations

To evaluate the performance of the tool with different choices of simulation parameters, we test the tool on two types of coupled toggle-switch (CTS) circuits (Fig. 1b, see Additional file 1: SI section “Results” for mathematical models). They both contain several toggle-switch motifs, but different connecting patterns among these motifs, where the type I circuits (CTS-I) have unidirectional activations among A genes (B genes), while the type II circuit (CTS-II) have mutual activations among A genes (B genes). These circuits have been actively studied to understand the coupled cellular decision-making processes [37, 38]. By changing the number of toggle-switch motifs, we can easily test RACIPE on circuits of different sizes. For each circuit, we generate 10,000 random models and solve steady-state expressions starting from 1000 initial conditions for each model. As shown in Fig. 1b, for both types of circuits, the average simulation time to solve a RACIPE model scales linearly with the total number of parameters in the model, suggesting its potential use on large circuits. Of note, the total time to simulate all RACIPE models depends on other factors (the number of models, the number of initial conditions, etc.), which will be discussed in the next section.

### Convergence test

As mentioned above, there are two important simulation parameters - the number of RACIPE models (nRM) and, for each model, the number of initial conditions (nIC) that are used to find all possible stable steady states. When nRM and nIC are too small, the results from the ensemble of models may not converge and be statistically significant. However, having too large nRM and nIC sacrifices computational efficiency.

**Fig. 2:**
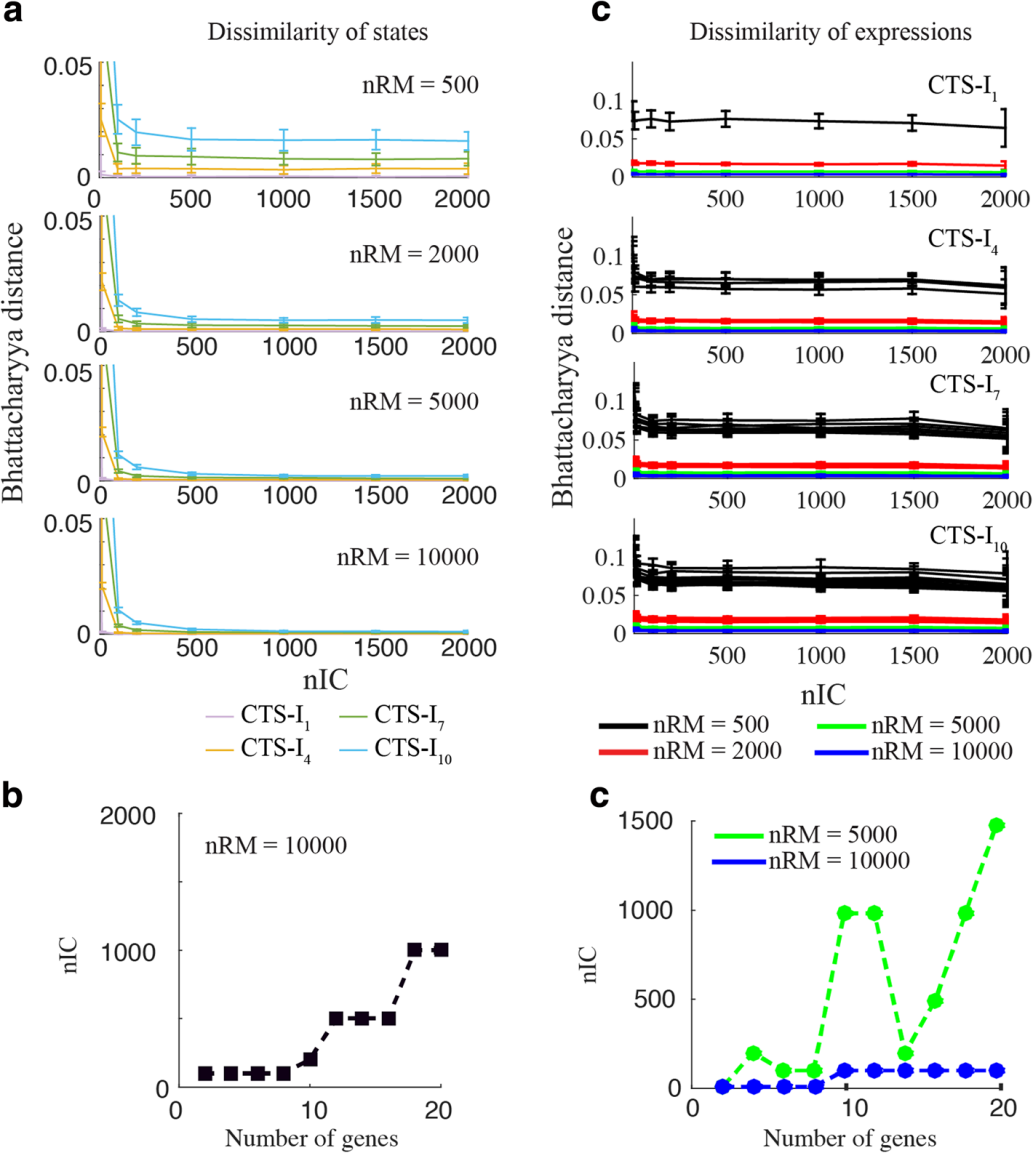
The effect of the number of initial conditions on the convergence of the RACIPE results. **a** For each coupled toggle-switch I (CTS-I) circuit (curves in different colors), the convergence is evaluated by the dissimilarity of states using different numbers of initial conditions (nIC in x-axis) and different numbers of RACIPE models (nRM in different panels). **b** The minimum nIC to get the converged distribution of the number of stables states when nRM equals 10,000. Different points represent the CTS-I circuits of different sizes. The minimum nIC is selected if the decrease of the Bhattacharyya distance is smaller than the threshold (0.0005, see Additional file 1: Figure S3) when nIC increases. **c** For each CTS-I circuit, the convergence is alternatively evaluated by the dissimilarity of expressions of each gene. Only the Ai genes for each circuit are plotted (one line per gene) and colored differently for different nRMs. The dissimilarity is less sensitive to nIC, but is dramatically reduced with the increase of nRM. **d** The minimum nIC to get the converged distribution of expressions. The minimum nIC is selected if the decrease of the Bhattacharyya distance is smaller than the threshold (0.0005, see Additional file 1: Figure S6) when nIC increases. nRM needs to be larger than 5000, otherwise the distribution is not converged even with nIC = 2000

**Fig. 3:**
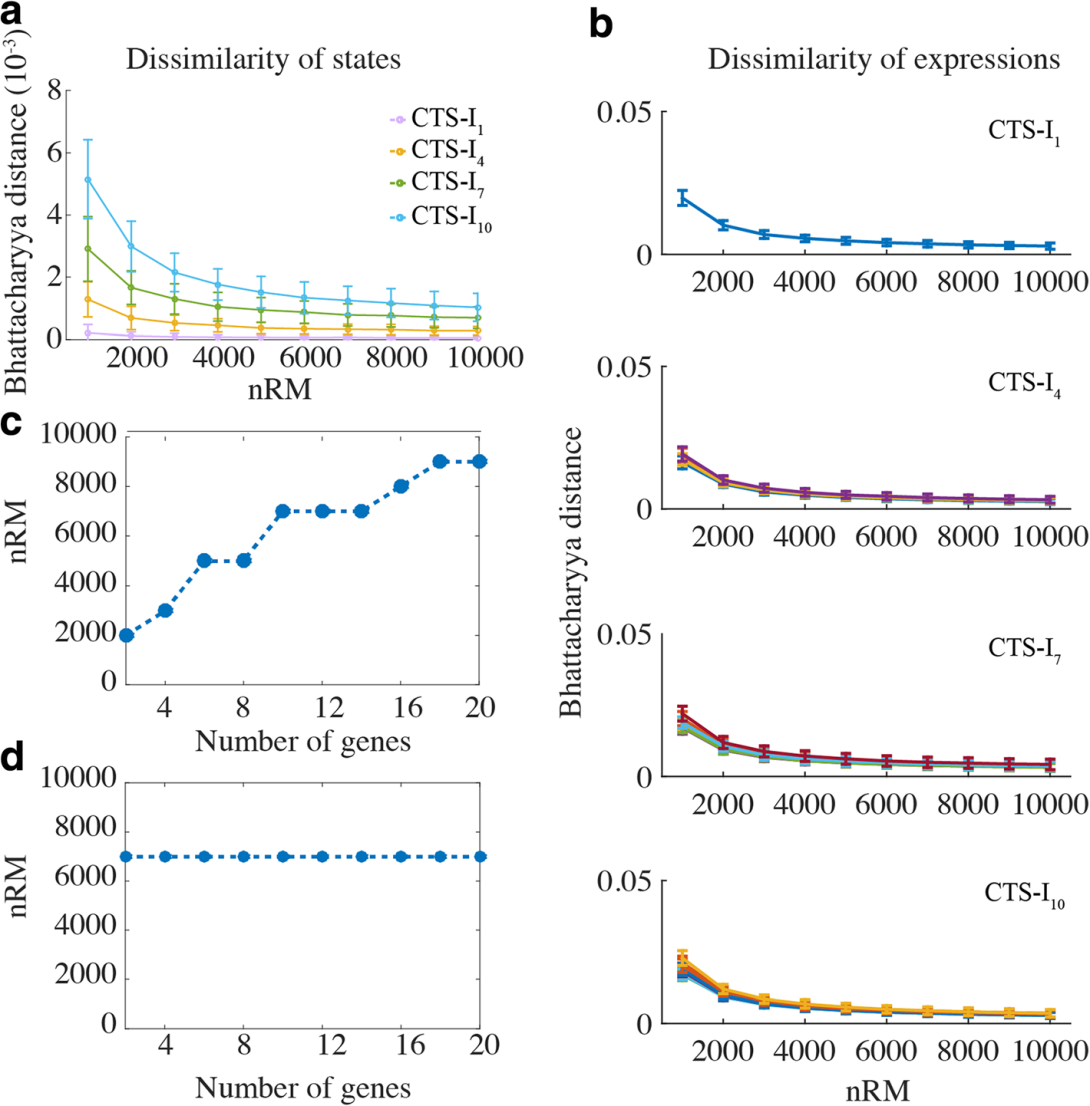
The effect of the number of RACIPE models on the convergence of the results. **a** The dissimilarity of states as a function of nRM when nIC is 1000. **b** The dissimilarity of expressions as a function of nRM when nIC is 1000. **c** The minimum nRM as the function of the number of genes in each circuit. **d** The minimum nRM to get the converged distribution of gene expressions

To identify an optimal choice of nRM and nIC, we test the effects of both on the convergence of the simulation results by calculating the dissimilarity of the probability distribution of the number of stable states (referred to as the “dissimilarity of states”) and the distribution of gene expressions (referred to as the “dissimilarity of expressions”) using different values of nRM and nIC (Figs. 2 and 3). If the simulation results converge well, the dissimilarity values are expected to be small.

For every choice of nIC and nRM, we repeat the RACIPE calculations for ten times for each circuit and measure the dissimilarity of the above-mentioned probability distributions by the Bhattacharyya distance [39] ${D}_B=-\ln \Big({\sum}_{x\in X}\sqrt{p(x)q(x)}$, where *p* and *q* are two distributions. If the two distributions are exactly same, *D*_*B*_ equals to 0; The more different the two distributions are, the larger *D*_*B*_ becomes. We have also calculated the dissimilarity using a different distance metric (the Kullback–Leibler divergence [40]) and obtained similar results (Additional file 1: Figure S9).

To explore the effects of nRM on the distribution of the number of stable states, we repeat RACIPE on the circuit for ten times for a certain nRM, and calculate the distribution of the number of stable states for each replica. Then we compare the dissimilarity of the distributions (i.e. the dissimilarity of states) for different nRMs by calculating the average Bhattacharyya distances:

3 $$ {D}_B=\frac{1}{100}\sum \limits_{j=1}^{10}\sum \limits_{h=1}^{10}-\ln \left(\sum \limits_{x\in X}\sqrt{p_{n_ij}(x){p}_{n_mh}(x)}\right)\kern0.75em , $$

where ${p}_{n_ij}(x)$stands for the probability of the circuit with *x* number of stable states for a random model for a replica *j* when nRM equals to *n*_*i*_. *n*_*m*_ is the maximum nRM used in the test. Here, we fix *n*_*m*_ to 10,000. Similarly, we can explore the effects of nRM on the distribution of gene expressions. Similar approach is used to analyze the effects of nIC.

As shown in Fig. 2a and Additional file 1: Figures S3 and S4, the dissimilarity of states decreases when more initial conditions are used. When nIC is larger than 500, RACIPE can effectively identify most stable steady states, except for some rare states (the probability to be observed is less than 1%). To get converged distribution of the number of stable states, the minimum required nIC increases with the size of the circuit (Fig. 2b and Additional file 1: Figure S3). Surprisingly, the convergence of the distribution of expressions seems to be less sensitive to nIC (Fig. 2c and Additional file 1: Figure S5 and S6), as similar results are obtained no matter how small or larger nICs are selected. As suggested from Fig. 2d, with more than 10,000 RACIPE models, 100 initial conditions are sufficient to get converged results.

However, nRM has a significant influence on the convergence of the simulation results. From Fig. 2a and Additional file 1: Figure S4, increasing nRM dramatically lowers the dissimilarity of states. Also, without enough RACIPE models, the distribution of expressions does not converge even when a large nIC is used (Fig. 2d). Furthermore, when nIC equals to 1000, both the dissimilarity of states and gene expressions decrease when nRM increases (Fig. 3a, b and Additional file 1: Figure S8). To get converged results for the distribution of states, the minimum required nRM again increases with the size of the circuit (Fig. 3c and Additional file 1: Figure S10). However, the minimum required nRM to get the converged distribution of expressions is likely independent to the size of the circuit as long as it is more than 7000 (Fig. 3d). Interestingly, when the dissimilarities of states for different circuits are scaled by the maximum number of stable states of the circuits, the curves of the dissimilarities for each circuit overlap with each other (Additional file 1: Figure S8b). The results suggest that the higher dissimilarity of a larger circuit is due to the higher complexity of the system.

### Analysis of the RACIPE-generated data

**Fig. 4:**
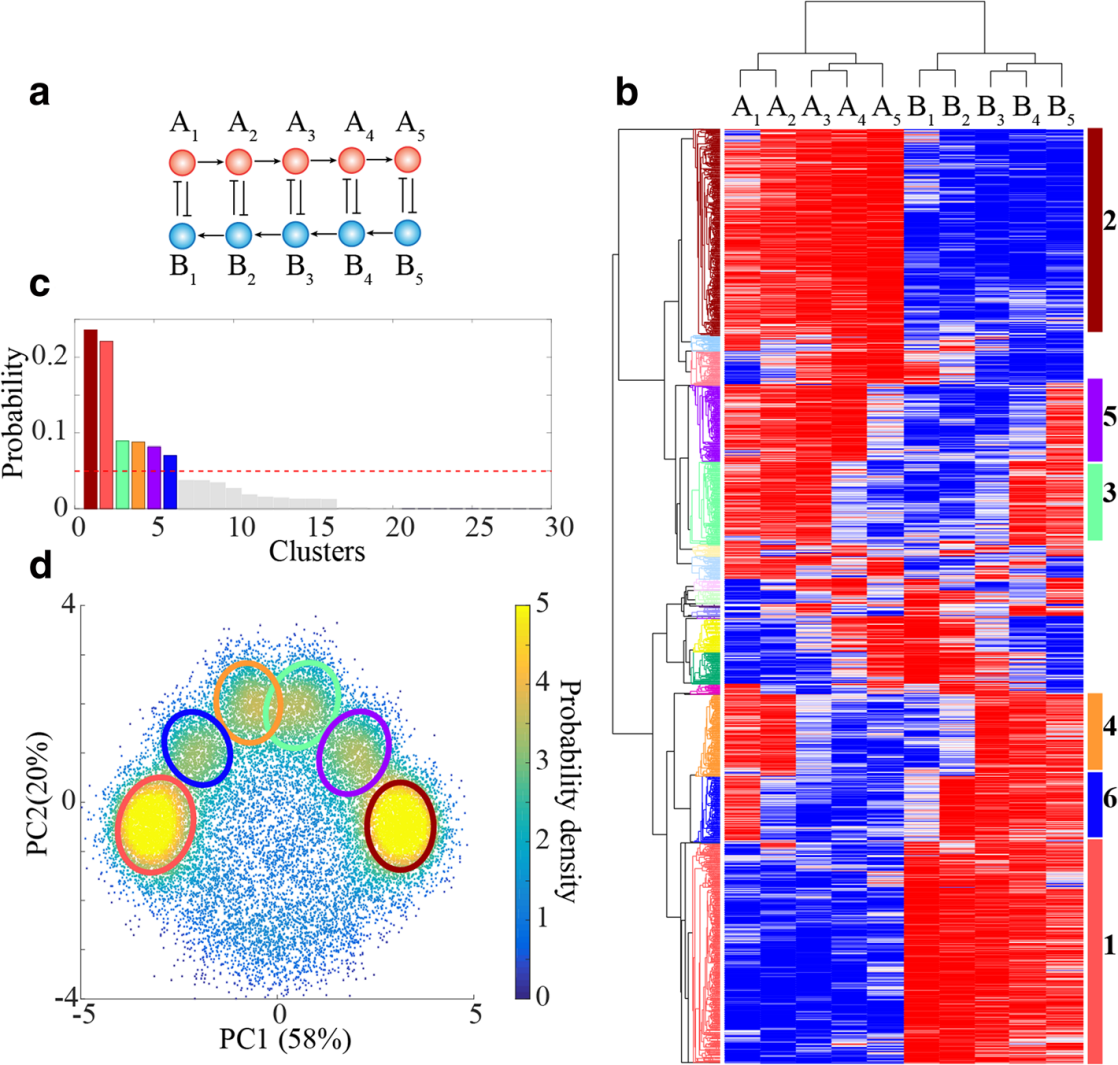
RACIPE identifies robust gene states of a coupled toggle-switch (CTS-I5) circuit. **a** Diagram of the CTS-I_5_ circuit. **b** Average linkage hierarchical clustering analysis of simulated gene expressions reveals six major clusters of distinct expression patterns. Each column corresponds to a gene, and each row corresponds to a stable steady state from a RACIPE model. **c**. Histogram of the fraction of gene expressions in each cluster. The cutoff is selected at 5% (Red dash line). **d** 2D probability density map of the RACIPE-generated gene expression data projected on to the first two principal components. The six gene clusters are highlighted by the same colors as those in (**b**)

Once RACIPE generates, for each model, the kinetic parameters and the stable-state gene expressions, a variety of statistical methods can be applied to analyze the data from the ensemble of models. In the following, we will illustrate these analyses in the context of a coupled toggle-switch circuit (CTS-I_5_, with five toggle switches) (Fig. 4a). We generate 10,000 RACIPE models, each of which is simulated starting from 1000 initial conditions. For each model, the maximum number of stable steady states is seven (Additional file 1: Figure S2); from 10,000 RACIPE models, there is a total of 24,425 steady states. These states could be regarded as the gene expressions of cells in a system obeying these dynamics.

To analyze the simulated gene expression, RACIPE utilizes average linkage hierarchical clustering analysis (HCA) using Euclidean distance after normalization of the expressions (see Additional file 1: SI 1.5–1.8 for details). From the heatmap (Fig. 4b), we observe six major clusters each of which has at least 5% fraction (Fig. 4c). The six major clusters, denoted by “gene states” below, are further confirmed by projecting all steady state solutions onto the first two principal components (PC1 and PC2) (Fig. 4d). From HCA, genes with similar functions are also grouped together. Strikingly, the gene expression patterns of the couple toggle-switch circuits, from the top to the bottom, correspond to a cascade of flips of the state of each toggle-switch motif (Fig. 4b). For instance, compared with gene state 2, gene state 5 has a flipped state in the fifth toggle-switch motif (A_5_ and B_5_).

**Fig. 5:**
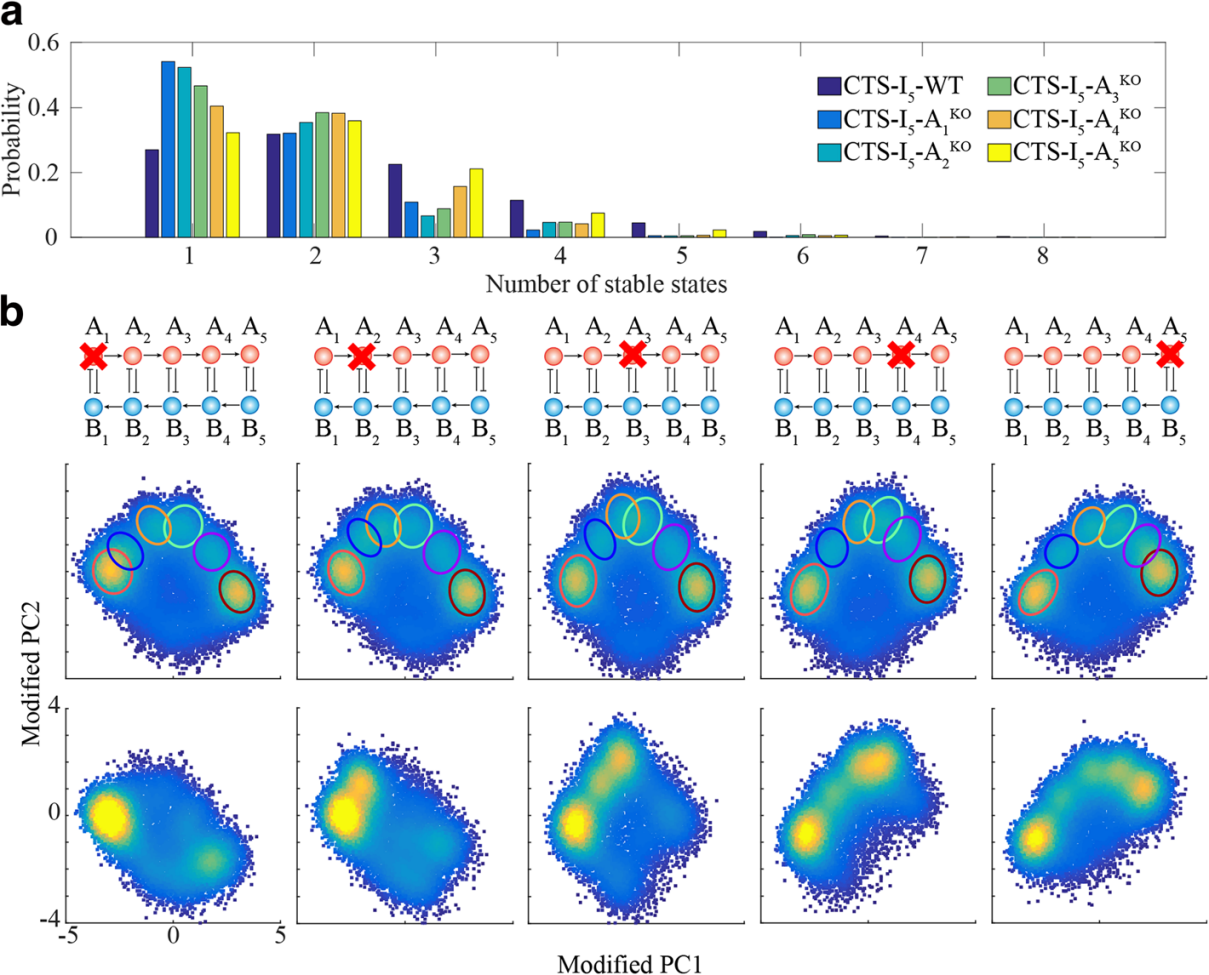
Perturbation analysis. **a** Probability distribution of the number of stable steady states of each model. Different colors represent the results of the complete circuit (CTS-I_5_-WT) and different knockout versions (CTS-I_5_-A_i_^KO^) analyzed by RACIPE. **b** Probability density maps of the RACIPE gene expressions projected on to the first two principal components. Note, for the knockout cases, the principal components are modified to reflect the zero expressions for the corresponding genes (see SI for details)

Moreover, RACIPE can identify the roles of individual genes in the dynamic behaviors of the circuit by in silico gene knockouts, one gene at a time (Fig. 5 and Additional file 1: Figure S13). Knocking out gene A_1_ dramatically changes the probability distribution of the number of stable states and probability distribution of gene expressions, while knocking out gene A_5_ leads to a similar distribution of the number of stable states and only one gene state is missing. Therefore, we find that, for coupled toggle-switch circuits, the importance of A_i_ genes gradually decreases - A_1_ is the most critical one and A_5_ is the least important one. Similarity, the importance of B_i_ genes is in the reverse order. In addition, RACIPE can identify the significantly differentiated parameters between two states by the statistical analysis of model parameters (Additional file 1: Figures S14, see SI 1.9), which further helps to elucidate the functions of gene circuits.

### Application to a B-lymphopoiesis gene circuit

The above example, while instructive, is only based on simple circuit motifs. To further evaluate the use of RACIPE, we analyze the properties of a gene regulatory circuit governing B-lymphopoiesis. This circuit was previously proposed by Salerno et al. [33] and analyzed mainly by traditional nonlinear dynamics methods, such as bifurcation analysis. Here we compare the RACIPE-generated gene expression data with microarray gene expression profiles of B cells from the previously published work by van Zelm et al. [34].

**Fig. 6:**
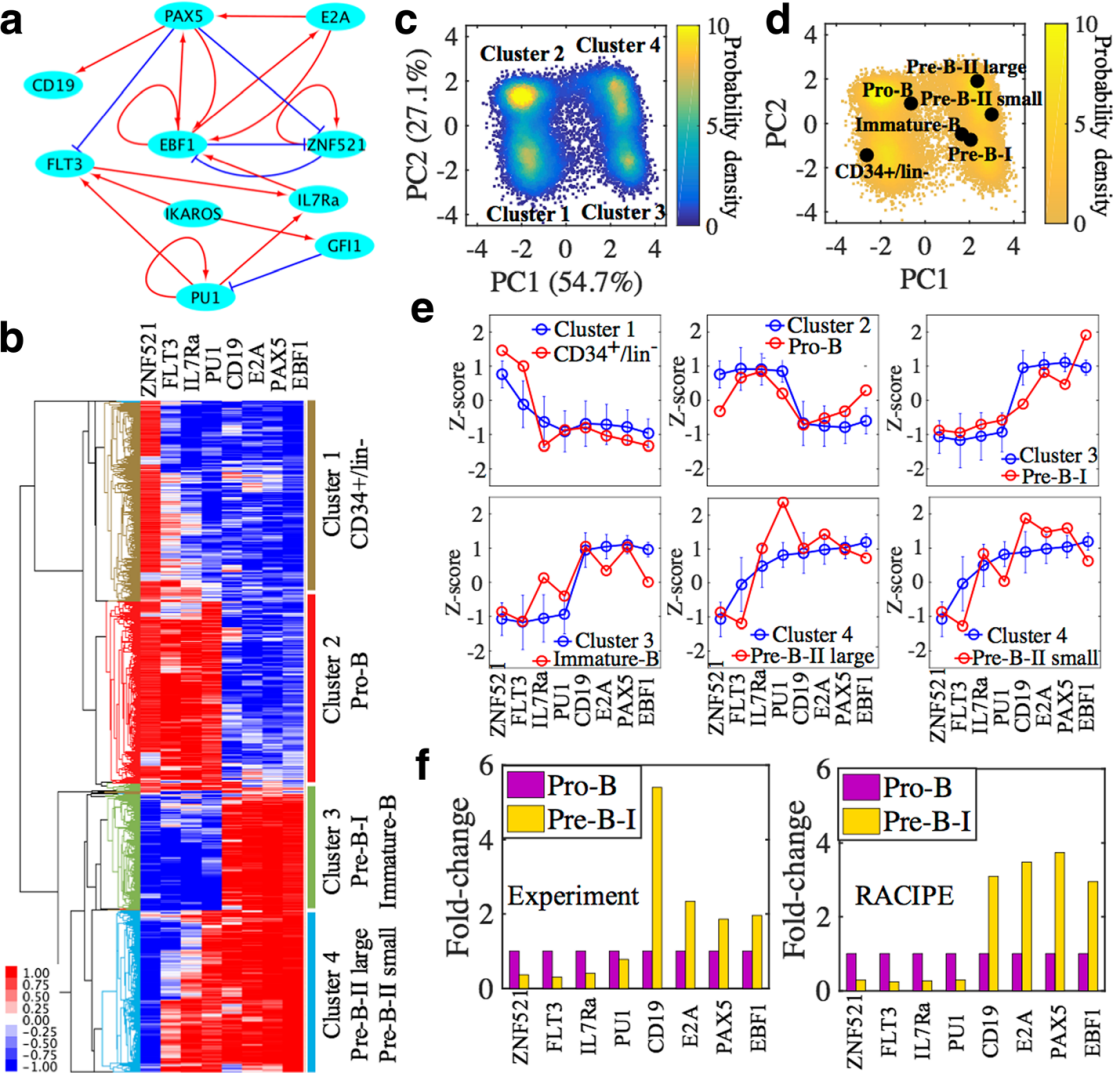
RAICPE identifies multiple gene expression states during B cell development. **a** A proposed gene regulatory circuit governing B-lymphopoiesis, adopted from (Salerno et al., 2015). The network consists of 10 transcription factors (TFs). Red arrows represent transcriptional activation and blue bar-headed arrows represent transcriptional inhibition. **b** Average linkage hierarchical clustering analysis of the gene expression data from all the RACIPE models using the Euclidean distance. Each column corresponds to a gene, and each row corresponds to a stable steady state. Four major gene states (clusters) are identified. **c** 2D probability density map of the RACIPE-predicted gene expression data projected on to the first two principal component axes. **d** The microarray expression profiling of different stages during B cell development (van Zelm et al., 2005) projected on to the same axes as shown in (**c**) (See Additional file 1: SI 1.10). **e** Comparison between experimental gene expression of various stages with in silico clusters. Blue dots and red dots represent the Z-scores of genes from the RACIPE models and experiments, respectively. Error bar for each blue dot represents standard deviation of the RACIPE-generated gene expression values. **f** Comparison between experimental gene expression fold-change from stage Pro-B to stage Pre-B-I with the computed fold-change by RACIPE

B cells that develop in the bone marrow progress through the multipotent progenitor (characterized by CD34^+^/lin^−^), pro-B, pre-B-I and pre-B-II large, pre-B-II small and immature-B stages sequentially [34]. The regulatory circuitry for lineage specification of hematopoietic multipotent progenitors is still not well understood. To address this issue, Salerno et al. constructed a gene regulatory circuit (Fig. 6a) governing B-lymphopoiesis based on literature search and confirmed the important role of ZNF521 (zinc finger protein 521) and EBF1 (Early B-Cell Factor 1) during the specification of B cells from the multipotent progenitor stage (CD34^+^/lin^−^) to the pro-B stage [33]. Here, we apply RACIPE to the same gene circuit and study the predicted gene expression patterns and how they are associated with various stages during B cell development.

Additional file 1: Figure S15 shows 10,000 models are good enough to capture the robust behaviors of the gene network for B-lymphopoiesis. The stable steady states from all models form four major clusters, which correspond to the stages CD34^+^/lin^−^, pro-B, (pre-B-I, Immature-B) and (Pre-B-II large, small), respectively (Fig. 6b-d). We further compare the microarray gene expression profiles with data generated by RACIPE. Even through there is only one sample in each stage from [34], the trend of the gene expression predicted by RACIPE agrees well with that from experiments, especially the comparison between cluster 1 and the CD34^+^/lin^−^ stage and that between cluster 3 and the Pre-B-I stage (Fig. 6e). From the hierarchical clustering analysis (Fig. 6b), we observe that there is a ‘switch-like’ change in the gene expression pattern from the stage pro-B to pre-B-I, as also shown in Fig. 6c. To test the prediction, we extract the microarray data of pro-B and pre-B-I and analyze the fold-change of the regulators in the circuit. Strikingly, the microarray data show the down-regulation of TF ZNF521, FLT3, IL7Ra and PU.1 and up-regulation of CD19, E2A, PAX5 and EBF1, which validates the prediction from the RACIPE analysis (Fig. 6f). In summary, RACIPE is able to provide a rich source of information from the regulatory circuit of B-lymphopoiesis and potentially capture the gene expression features of various stages during B cell development.

Although we observe agreement between in silico clusters by RACIPE and microarray data of various stages in B cell development, we might not yet be able to generate all information regarding the paths of B cell development. The reasons are at least two-fold. First, the result by RACIPE is highly dependent on the topology of the gene circuit and there might be important genes/regulations missing in the current circuit due to insufficient knowledge from available data. Second, due to the very limited number of experimental samples, i.e., one in each stage, the comparison with clusters by RACIPE might be inaccurate. However, with even the limited information, RACIPE has been shown to capture the change of multiple master regulators across various stages during B cell development. Further studies including construction of a more complete regulatory circuit for B cell development and measures of gene expression of more samples at various stages are needed to fully understand the state transitions of B cell progression.

## Discussion

In this study, we introduced a new tool based on our recently developed computational algorithm, named *ra*ndom *ci*rcuit *pe*rturbation (RACIPE). The tool is built in C and will be freely available for public use. Compared to the randomization approaches to generate benchmark datasets for network inference [41, 42], RACIPE features a unique "half-functional" rule to carefully sample the parameter space. In addition, RACIPE can identify the most robust features of a gene circuit, such as gene expression clusters, without the need to know detailed values of kinetic parameters.

To better understand the performance of RACIPE, we particularly explored the effects of two key simulation parameters, the number of initial conditions (nIC) and the number of RACIPE models (nRM), on the convergence of the statistical analysis. Insufficient nIC and nRM may lead to inconsistent results in the repeats of the same simulation. Figs. 2 and 3 are good references for an initial guess of these parameters and users can always identify the optimal nIC and nRM with a similar analysis. From our tests, the time cost of the RACIPE tool scales linearly with the total number of parameters used in the mathematical model, suggesting its potential use in analyzing large gene networks.

To illustrate the use of RACIPE, we applied it to a coupled toggle-switch (CTS-I_5_) circuit consisting of five toggle switches, a circuit that has an implication in coupled decision-making of multiple cell fates. From the RACIPE-generated expression data, we identified six major clusters by both HCA and PCA. In addition, we analyzed the role of each gene on circuit dynamics by in silico gene knockout (Fig. 5). To further show the predictive power of RACIPE, we applied it on a published B-lymphopoiesis gene regulatory circuit. The gene expression patterns of various stages during B cell development can be efficiently captured by RACIPE. Notably, the fold-change of master regulators from stage ‘Pro-B’ to stage ‘Pre-B-I’ predicted by RACIPE agrees well with that from the microarray data. These results show that RACIPE can not only reveal robust gene expression patterns, but also help uncover the design principle of the circuit.

The capability of RACPE in identifying circuit functions using a randomization approach reinforces the hypothesis that circuit dynamics are mainly determined by circuit topology [43] not by detailed kinetic parameters. Indeed, it is commonly believed that, through evolution, gene circuits of important pathways should be robustly designed to be functional [14] even in a dynamic and heterogeneous environment [44]. In RACIPE, we take advantage of this feature to interrogate the robustness of a gene circuit by randomly perturbing all the kinetic parameters, from which we evaluate the most conserved properties.

Although we believe RACIPE has wide applications in systems biology, there are a few limitations of the current version. First, while all parameters are completely randomized to generate models, some of these models might not be realistic because some parameters are unlikely to be perturbed in cells, such as the number of binding sites. In these cases, incorporating relevant experimental evidences will improve the modeling. Second, RACIPE is unique in generating data of both gene expression and model parameters. Although we have shown that the parameters in models from different gene state clusters are distinct (Additional file 1: Figure S14), further data analysis methods are needed to fully understand the roles of each parameter in circuit behavior. Third, the current RACIPE only models regulatory circuits of transcription factors. However, the same approach can be extended to model biological pathways, which typically involves multiple types of regulation, such as protein-protein interactions and microRNA-mediated regulations. Fourth, we currently use deterministic ODE-based method to simulate the circuit dynamics. Since gene expression noise has been shown to play crucial roles in circuit dynamics [45, 46], it is important to extend the method to stochastic analysis. Lastly, the quality of the circuit topology may dramatically impact the quality of RACIPE modeling. An accurate inference method for constructing gene circuits is especially important. To associate the parameters with network dynamics, a global sensitivity analysis [47, 48] and hyperparameter optimization will be especially useful to measure the effects of each parameter and fit RACIPE models into real gene expression data. Further improvements on these aspects will greatly improve the usability of this randomization-based approach and contribute to a better understanding of the operative mechanisms of gene regulatory circuits.

## Conclusions

In this paper, we have presented a new computational tool based on our recently developed computational method, RACIPE. By taking the topology of GRNs as the only inputs, RACIPE can unbiasedly generate an ensemble of mathematical models, each of which is characterized by a unique set of parameters. We evaluated the convergence of RACIPE-generated results by tuning two simulation parameters – number of initial conditions (nIC) and number of RACIPE models (nRM). In addition, we applied RACIPE on the coupled toggle-switch circuits and a published B-lymphopoiesis network to illustrate the statistical methods that can be applied to RACIPE-generated data. All told, we expect RACIPE to pave a new way for the community to explore the robust functions of gene regulatory circuits with the insufficient knowledge of the kinetic parameters.

## Availability and requirements

Project name: RACIPE.

Project home page: https://github.com/simonhb1990/RACIPE-1.0

Operating system(s): Platform independent.

Programming language: C.

Other requirements: None.

License: Apache 2.0.

Any restrictions to use by non-academics: None.

## Additional file

Additional file 1: Supplementary algorithmic details and user guide of RACIPE and supplementary figures. (DOCX 6198 kb)

## Abbreviations

CTS: Coupled toggle-switch
EMT: Epithelial-to-mesenchymal transition
GRN: Gene regulatory network
HCA: Hierarchical clustering analysis
nIC: Number of initial conditions
nRM: Number of RACIPE models
ODE: Ordinary differential equation
PCA: Principal component analysis
RACIPE: Random circuit perturbation
