# Supplementary material for: RACIPE: a computational tool for modeling gene regulatory circuits using randomization
Source: BMC Syst Biol. 2018 Jun 19;12:74. doi: 10.1186/s12918-018-0594-6 (PMC6006707; doi:10.1186/s12918-018-0594-6)
Supplement: Supplementary file 1 — Supplementary algorithmic details and user guide of RACIPE and supplementary figures. (DOCX 6198 kb) [file 12918_2018_594_MOESM1_ESM.docx]

RACIPE: A Computational Tool for Modeling Gene Regulatory Circuits using Randomization

Bin Huang^1^, Dongya Jia^1,2^, Jingchen Feng^1^, Herbert Levine^1,3,4,5,*^, José N. Onuchic^1,4,5,6,*^, Mingyang Lu^7,*^

^1^Center for Theoretical Biological Physics, Rice University, Houston, TX, United States of America

^2^Program in Systems, Synthetic and Physical Biology, Rice University, Houston, TX, United States of America

^3^Department of Bioengineering, Rice University, Houston, TX, United States of America

^4^Department of Biosciences, Rice University, Houston, TX, United States of America

^5^Department of Physics and Astronomy, Rice University, Houston, TX, United States of America

^6^Department of Chemistry, Rice University, Houston, TX, United States of America

^7^The Jackson Laboratory, Bar Harbor, ME, United States of America

*To whom correspondence should be addressed.

**Supplementary Data**

**Table of contents**

1. **Algorithmic details of Random Circuit Perturbation (RACIPE)**

**1.1 Defining the ranges of parameters for randomization**

**1.2 Numerically solving the nonlinear dynamics of a RACIPE model**

**1.3 Finding all possible stable states**

**1.4 Perturbation analysis**

**1.5 Normalization of gene expression data**

**1.6 Clustering analysis**

**1.7 The probability distribution of the number of stable states and gene expression**

**1.8 The probability to find all the stable states for a RACIPE model given a certain number of initial conditions (nIC)**

**1.9 Comparison of the parameters between two gene states.**

**1.10 Comparison between experimental microarray data and *in silico* clusters**

1. **Mathematical models of coupled toggle-switch (CTS) circuits**

**2.1 Coupled toggle-switch I (CTS-I) with n toggle switches**

**2.2 Coupled toggle-switch II (CTS-II) with n toggle switches**

1. **Supplementary Figures**
2. **Usage Guide for RACIPE (also available of the README file)**

**1. Algorithmic details of Random Circuit Perturbation (RACIPE)**

**1.1 Defining the ranges of parameters for randomization**

The RACIPE method generates an ensemble of models with different set of parameters, which are randomly sampled in a certain range following a certain distribution. The tool provides options to choose from three types of distributions: uniform distribution, rectified Gaussian distribution (its negative elements is reset to zero) and exponential distribution. Users can select one of them using the *“-dist”* option. The default ranges of parameters for randomization are as follows:

| Parameters | Min to Max Values  (Uniform) | Mean, Standard Deviation  (Rectified Gaussian)^+^ | Mean  (Exponential) |
| --- | --- | --- | --- |
| Maximum production rate () | 1-100 | 50.5, 49.5 | 50.5 |
| Degradation rate () | 0.1-1 | 0.55, 0.45 | 0.55 |
| Fold change ()^*^ | 1-100 | 50.5, 49.5 | 50.5 |
| Threshold ($X_{0}$) | The ranges, which depend on the inward regulations,  are estimated by a Monte Carlo simulation. | | |
| Hill coefficient ()^#^ | 1-6 | 3.5, 2.5 | 3.5 |

**Table S1**. Default ranges of the parameters for randomization. The ranges of threshold levels are estimated by a mean-field approximation considering all the inward regulations. More details are available in our previous paper (Huang *et al.*, 2017).

^*^For Uniform distribution, the fold change of an inhibitory regulation ranges from 0.01 to 1. But the inverse of is uniformly sampled from 1 to 100 instead of sampling uniformly from 0.01 to 1. By doing so, we ensure that the average is about 0.046, instead of ~ 0.5. Similar approaches are adopted for the Gaussian and exponential distributions.

^#^The value of the Hill coefficient is an integer from 1 to 6.

^+^The negative part is trimmed.

**1.2 Numerically solving the nonlinear dynamics of a RACIPE model**

For a RACIPE model with a particular set of model parameters, we numerically solve the model by the Euler method. The simulation of the model stops either when it reaches the steady state or when it reaches the maximum steps, which is adjustable using *the “-maxiters”* option.

To thoroughly explore the solution space, we repeat the above simulations many times with different initial conditions, the number of which is defined by the *“-num_ode”* option. The initial level of each gene is randomly picked from a log-uniform distribution ranging from the minimum level to the maximum level of the gene. We can estimate the minimum and maximum levels by the following equations:

$$x_{min}=G_{x}(\prod_{i=1}^{n} \lambda_{i}^{-})/(k_{x}(\prod_{j=1}^{m} \lambda_{j}^{+}))$$

$$x_{max}=G_{x}/k_{x}$$

where $G_{x}$ is the maximum production rate and $k_{x}$ is the degradation rate of gene x. $\lambda_{i}^{-}$ stands for the fold change caused by the inhibitor i, and $\lambda_{j}^{+}$ stands for the fold change caused by the activator j.

**1.3 Finding all possible stable states**

From all of the stable steady state solutions of a RACIPE model using different initial conditions, we can identify distinct stable states, defined as those whose Euclidean distance of the levels among them are all larger than a certain threshold. The default value of the threshold is 1, which can be adjusted by the *“-thrd”* option.

**1.4 Perturbation analysis**

For a gene knockout simulation, RACIPE generates an ensemble of models by fixing the maximum production rate of the knockout gene to zero and randomizing the other the parameters in the same ranges as in the standard RACIPE. For a gene overexpression simulation, RACIPE generates an ensemble of models by fixing the production rate of the overexpressed gene to the maximum and randomizing the other parameters.

We compare the simulations from a knockout condition (refer to as treatment (T) models) with those from normal conditions (refer to as wide type (WT) models) as follows (Fig. 5b). First, using the RACIPE gene expression data from the normal condition, we compute the first two principal components (PCs) (Fig.4d). Second, for the corresponding knockout treatment, we modify the PCs by setting the loading coefficient of the knockout gene to zero. Third, we then project the RACIPE models from the knockout condition to the modified PCs.

**1.5 Normalization of gene expression data**

Following the standard procedure for processing experimental gene expression, we preprocess RACIPE predicted gene expression data by a standard normalization method. The gene expression levels from the WT models $x_{i}^{WT}$ are normalized by log transformation and then standardization, i.e.

$$x_{i}^{WT}\to\frac{{log}_{2}\left( x_{i}^{WT} \right)-\overline{{log}_{2}(x_{i}^{WT})}}{{\sigma(log}_{2}(x_{i}^{WT}))}$$

The gene expression levels from the treatment models $x_{i}^{T}$ is normalized by log transformation and then standardization by using the mean ($\overline{{log}_{2}(x_{i}^{WT})}$) and the standard deviation (${\sigma(log}_{2}(x_{i}^{WT}))$) of the WT models, i.e.

$$x_{i}^{T}\to\frac{{log}_{2}\left( x_{i}^{T} \right)-\overline{{log}_{2}(x_{i}^{WT})}}{{\sigma(log}_{2}(x_{i}^{WT}))}$$

**1.6 Clustering analysis**

We perform clustering analysis on the normalized gene expression data from RACIPE models, as shown in Fig. 4b and Fig. 6. Each column represents a gene, and each row represents a stable steady state from a RACIPE model. For models with more than one stable state, the gene expression profiles for all the stable states are entered in multiple rows. We apply the average linkage hierarchical clustering analysis using Euclidean distance by Cluster 3.0 (de Hoon *et al.*, 2004) and the results are visualized by JavaTreeview (Saldanha, 2004). Principal component analysis can be performed on the same data using the “*pca*” function in Matlab/2014b (Fig. 4d, Fig. 6b and Fig. 7c). Major clusters of the expression data can be readily recognized from the probability density map projected on to the first two principal component axes.

**1.7 The probability distribution of the number of stable states and gene expression**

The probability distribution of the number of stable states is calculated by:

$$p\left( i \right)= \frac{n_{i}}{N}$$

where $n_{i}$ stands for the number of RACIPE models with i number of stable steady states, while N is the total number of RACIPE models.

The probability distribution of gene expression is calculated by:

$$p_{j}\left( x \right)= \frac{m_{x}^{j}}{M}$$

where $m_{x}^{j}$ stands for the number of stable steady states whose expression level of gene j is x, while M is the total number of stable steady states across all the RACIPE models.

**1.8 The probability to find all the stable states for a RACIPE model given a certain number of initial conditions (nIC)**

We firstly solve each RACIPE model i with 10,000 initial conditions to thoroughly find all the stable states, the total number of which is denoted as $N_{10000}^{i}$. With different nICs (x), we solve the same RACIPE models again to find the stable states, the total number of which is denoted as $N_{x}^{i}$. Therefore, for each model i, we can calculate the faction of stable states that are captured by the versions of RACIPE with different nICs, ${N_{x}^{i}}/{N_{10000}^{i}}$. Lastly, we calculate the probability to find all the stable states for a RACIPE model with a certain nIC, and its mean and standard deviation (Fig. S6).

**1.9 Comparison of the parameters between two gene states.**

Each parameter from all of the RACIPE models is first standardized by subtracting its mean and dividing the difference by its standard deviation. Then, we find the RACIPE models (group I) that have stable states from the gene state cluster i but no states from the cluster j. Similarly, we search for the RACIPE models (group J) that have stable states from the gene state cluster j but no states from the cluster i. The means of each parameter in both groups are plotted in a 2D diagram (Fig. S12). Note that, for inhibitory regulation, the fold changes (typically less than 1) are inversed before the statistical analysis (including the normalization step).

**1.10 Comparison between experimental microarray data and *in silico* clusters**

The gene expression data of ZNF521, FLT3, IL7Ra, PU.1, CD19, E2A, PAX5 and EBF1 for each stage during B cell development were extracted from (van Zelm *et al.*, 2005). If there are multiple probes for the same gene, the probe with the maximum expression level was selected. To project the microarray data to the PC1 and PC2 generated by RACIPE (Figure 6D), we calculated the z-score of each gene across all 6 experimental samples, representing CD34+/lin-, Pro-B, Immature-B, Pre-B-I, Pre-II large and Pre-II small respectively.

To avoid the effect of different cluster sizes, we normalized the microarray data for each gene by the weighted mean and weighted standard deviation as follows,

$$\bar{x_{i}^{w}}=\sum_{j=1}^{6} w_{j}x_{i}^{j}$$

$$\bar{\sigma_{i}^{w}}=\sqrt{\sum_{j=1}^{6} w_{j}{(x_{i}^{j}-\bar{x_{i}^{w}})}^{2}}$$

where $\bar{x_{i}^{w}}$ and $\bar{\sigma_{i}^{w}}$ represent the weighted mean and weighted standard deviations for gene $i$, respectively. $x_{i}^{j}$ represents the expression level of gene $i$ in sample $j$ and $w_{j}$ represents the weighting factor for the sample $j$. The weighting factor is determined by the corresponding cluster size. The weighting factors for CD34+/lin-, Pro-B, Pre-B-I, Pre-II large, Pre-II small and Immature-B, are [0.31, 0.27, 0.17/2, 0.25/2, 0.25/2, 0.17/2]. Since both stage Immature-B and Pre-B-I correspond to cluster 3, both stage Pre-II large and Pre-II small correspond to cluster 4, the weighting factor for each of these stages is half of the size of the corresponding clusters.

**2. Mathematical models of coupled toggle-switch (CTS) circuits**

**2.1 Coupled toggle-switch I (CTS-I) with n toggle switches:**

$$\dot{A}_{1}=G_{A_{1}}H^{S}\left( B_{1},{B_{1}}_{A_{1}}^{0},n_{B_{1}A_{1}},\lambda_{B_{1}A_{1}}^{-} \right)-k_{A_{1}}A_{1}$$

$\dot{A}_{i}=G_{A_{i}}H^{S}\left( B_{i},{B_{i}}_{A_{i}}^{0},n_{B_{i}A_{i}},\lambda_{B_{i}A_{i}}^{-} \right)H^{S}\left( A_{i-1},{A_{i-1}}_{A_{i}}^{0},n_{A_{i-1}A_{i}},\lambda_{A_{i-1}A_{i}}^{+} \right)/\lambda_{A_{i-1}A_{i}}^{+}-k_{A_{i}}A_{i}$ (i>1)

$\dot{B}_{j}=G_{B_{j}}H^{S}\left( A_{j},{A_{j}}_{B_{j}}^{0},n_{A_{j}B_{j}},\lambda_{A_{j}B_{j}}^{-} \right)H^{S}\left( B_{j+1},{B_{j+1}}_{B_{j}}^{0},n_{B_{j+1}B_{j}},\lambda_{B_{j+1}B_{j}}^{+} \right)/\lambda_{B_{j+1}B_{j}}^{+}-k_{B_{j}}B_{j}$ (j<n)

$$\dot{B}_{n}=G_{B_{n}}H^{S}\left( A_{n},{A_{n}}_{B_{n}}^{0},n_{A_{n}B_{n}},\lambda_{A_{n}B_{n}}^{-} \right)-k_{B_{n}}B_{n}$$

**2.2 Coupled toggle-switch II (CTS-II) with n toggle switches:**

$$\dot{A}_{1}=G_{A_{1}}H^{S}\left( B_{1},{B_{1}}_{A_{1}}^{0},n_{B_{1}A_{1}},\lambda_{B_{1}A_{1}}^{-} \right)H^{S}\left( A_{2},{A_{2}}_{A_{1}}^{0},n_{A_{2}A_{1}},\lambda_{A_{2}A_{1}}^{+} \right)/\lambda_{A_{2}A_{1}}^{+}-k_{A_{1}}A_{1}$$

$$\dot{A}_{i}=G_{A_{i}}H^{S}\left( B_{i},{B_{i}}_{A_{i}}^{0},n_{B_{i}A_{i}},\lambda_{B_{i}A_{i}}^{-} \right)H^{S}\left( A_{i-1},{A_{i-1}}_{A_{i}}^{0},n_{A_{i-1}A_{i}},\lambda_{A_{i-1}A_{i}}^{+} \right)$$

$H^{S}\left( A_{i+1},{A_{i+1}}_{A_{i}}^{0},n_{A_{i+1}A_{i}},\lambda_{A_{i+1}A_{i}}^{+} \right)/(\lambda_{A_{i-1}A_{i}}^{+}\lambda_{A_{i+1}A_{i}}^{+})-k_{A_{i}}A_{i}$ (1<i<n)

$$\dot{A}_{n}=G_{A_{n}}H^{S}\left( B_{n},{B_{n}}_{A_{n}}^{0},n_{B_{n}A_{n}},\lambda_{B_{n}A_{n}}^{-} \right)H^{S}\left( A_{n-1},{A_{n-1}}_{A_{n}}^{0},n_{A_{n-1}A_{n}},\lambda_{A_{n-1}A_{n}}^{+} \right)/\lambda_{A_{n-1}A_{n}}^{+}-k_{A_{n}}A_{n}$$

$$\dot{B}_{1}=G_{B_{1}}H^{S}\left( A_{1},{A_{1}}_{B_{1}}^{0},n_{A_{1}B_{1}},\lambda_{A_{1}B_{1}}^{-} \right)H^{S}\left( B_{2},{B_{2}}_{B_{1}}^{0},n_{B_{2}B_{1}},\lambda_{B_{2}B_{1}}^{+} \right)/\lambda_{B_{2}B_{1}}^{+}-k_{B_{1}}B_{1}$$

$$\dot{B}_{j}=G_{B_{j}}H^{S}\left( A_{j},{A_{j}}_{B_{j}}^{0},n_{A_{j}B_{j}},\lambda_{A_{j}B_{j}}^{-} \right)H^{S}\left( B_{j-1},{B_{j-1}}_{B_{j}}^{0},n_{B_{j-1}B_{j}},\lambda_{B_{j-1}B_{j}}^{+} \right)$$

$H^{S}\left( B_{j+1},{B_{j+1}}_{B_{j}}^{0},n_{B_{j+1}B_{j}},\lambda_{B_{j+1}B_{j}}^{+} \right)/(\lambda_{B_{j-1}B_{j}}^{+}\lambda_{B_{j+1}B_{j}}^{+})-k_{B_{j}}B_{j}$ (1<j<n)

$$\dot{B}_{n}=G_{B_{n}}H^{S}\left( A_{n},{A_{n}}_{B_{n}}^{0},n_{A_{n}B_{n}},\lambda_{A_{n}B_{n}}^{-} \right)H^{S}\left( B_{n-1},{B_{n-1}}_{B_{n}}^{0},n_{B_{n-1}B_{n}},\lambda_{B_{n-1}B_{n}}^{+} \right)/\lambda_{B_{n-1}B_{n}}^{+}-k_{B_{n}}B_{n}$$

where $G_{x}$ represents the maximum production rate of gene x, and $k_{x}$represents the degradation rate of gene x. For the regulatory link (either activation or inhibition) from gene x to gene y, $x_{y}^{0}$ represents the threshold level of gene x, $n_{xy}$is the Hill coefficient, and $\lambda_{xy}$ is the maximum fold change of the gene y level caused by gene x. $\lambda_{xy}<1$ represents an inhibitory regulation, referred to as $\lambda_{xy}^{-}$, while $\lambda_{xy}>1$ for an excitatory regulation, referred to as $\lambda_{xy}^{+}$. The circuit diagrams and the notation of each gene for the CTS-I and CTS-II motifs are shown in Fig. 1b.

3. **Supplementary Figures**


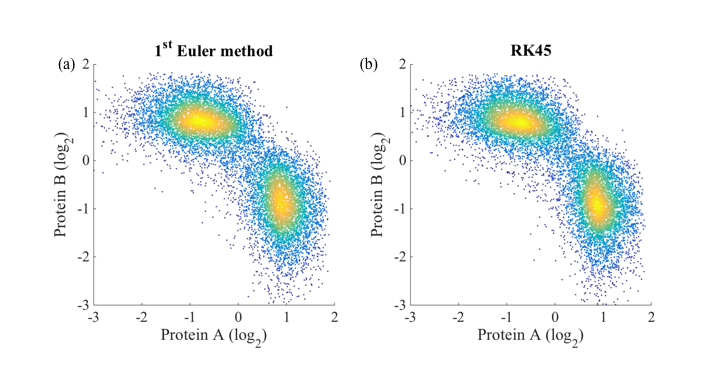


**Figure S1. 2D probability density map of the RACIPE-predicted gene expression data** **for the toggle-switch circuit.** The ODEs models are solved by either the 1^st^ Euler method (left panel) or the Runge–Kutta (RK45) method (right panel).

**Figure S2. Distribution of the number of stable states for the coupled toggle-switch (CTS-I) circuit.** For each circuit (different panels), the number of stable states is calculated from each RACIPE model. A distribution of the number of stable states is obtained by statistical analysis on an ensemble of 10000 models. This process is repeated for ten times to obtain the mean and standard deviation (error bars) of the distribution. To obtain the maximum number of stable states, we define it as the number of stable states (n, in x-axis) whose probability P(x=n) is no less than 5%. The black markers indicate this value, as it is the intersection of the probability distribution and a cutoff of 5% (red dashed line).

**Figure S3. The minimum nIC required for the convergence of RACIPE using the distribution of the number of stable states**. For each number of random models (nRM, different panels) and number of initial conditions for each model (nIC, different points), the distribution of the number of stable states (similar to those in Figure S2) is computed. The process is repeated for ten times to compute the average Bhattacharyya distance of the distributions (See Equation 3 in the Main Text). The y-axis shows the difference between the average Bhattacharyya distance at each nIC and that at 2000 nIC. The minimum nIC is selected if the difference is below a threshold (0.0005, black line). The result of the minimum nIC for each circuit is shown in Fig. 2b.

**Figure S4. The dissimilarity of states for each coupled toggle-switch I (CTS-I) circuit using different nICs** (1, 10, 100, 200, 500, 1000, 1500 and 2000) **and nRMs** (500 (a), 2000 (b), 5000 (c), 10000 (d))**.**


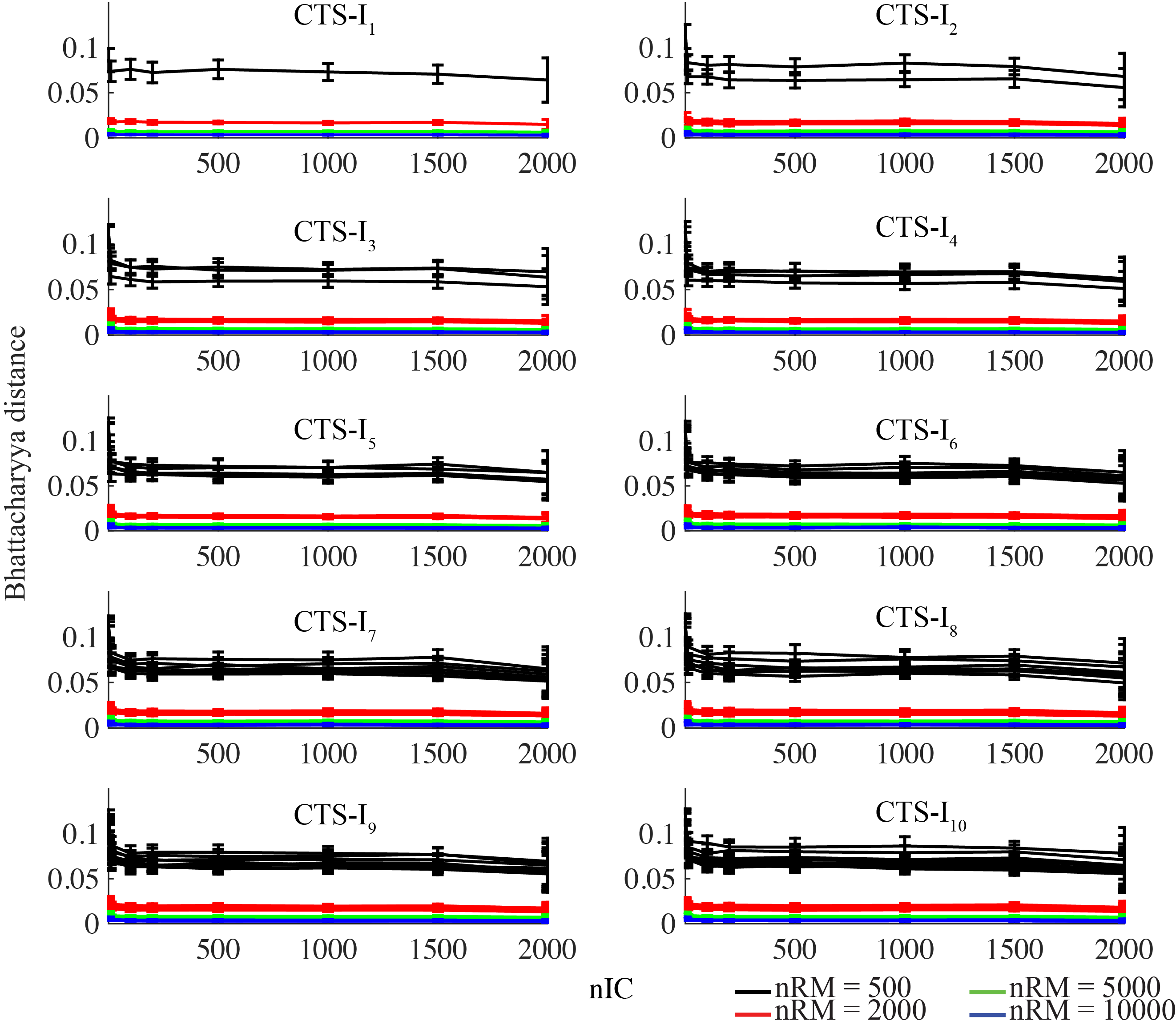


**Figure S5. The dissimilarity of expressions of each gene for each coupled toggle-switch I (CTS-I) circuit using different nICs** (1, 10, 100, 200, 500, 1000, 1500 and 2000) **and nRMs** (500, 2000, 5000, 10000). Due to the symmetry of each circuit, only the A_i_ genes for each circuit is plotted (each line stands for a gene) and colored differently for different nRMs.

**Figure S6. The minimum nIC required for the convergence of RACIPE using the distribution of gene expression.** For each number of initial conditions for each model (nIC, different points), the distribution of the expression of each gene is computed. The process is repeated for ten times to compute the average Bhattacharyya distance of the distributions (See Equation 1 in the Main Text). The y-axis shows the difference between the average Bhattacharyya distance at each nIC and that at 2000 nIC. Due to the symmetry of each circuit, only the A_i_ genes for each circuit is plotted (each line stands for a gene) and colored differently for different nRMs. The minimum nIC is selected if the difference is below a threshold (0.0005, orange line). The result of the minimum nIC for each circuit is shown in Fig. 2d.

**Figure S7. The probability of identifying all the stable states for a RACIPE model using different nICs.** **(a)** Each line stands for a specific multi-stable case, such as bi-stable or tri-stable models. **(b, c)** Rare stable states for each model are exclude if they have less than 0.1% (panel b) or 1% (panel c) chance to be observed with 10000 initial conditions.

**Figure S8.** **The dissimilarity of states as a function of nRM for each coupled toggle-switch (CTS-I) circuit.** **(a)** no scaling; **(b)** The dissimilarity values are scaled by the maximum number of the stable states for each circuit.

**
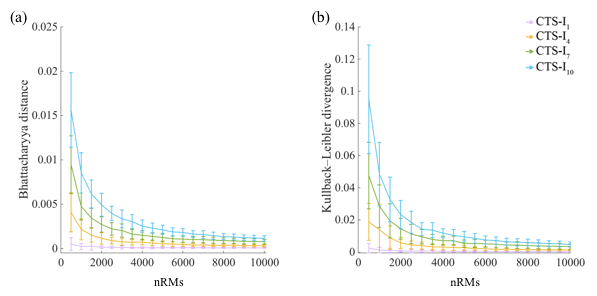
**

**Figure S9. The dissimilarity of states as a function of nRM for each coupled toggle-switch (CTS-I) circuit. (a)** The dissimilarity values are calculated by the Bhattacharyya distance; **(b)** The dissimilarity values are calculated by the Kullback-Leibler divergence.

**Figure S10.** **The minimum nRM required for the convergence of RACIPE using the distribution of the number of stable states.** For each number of random models (nRM, different points) of each circuit (CTS-I, different panels), the distribution of the number of stable states (similar to those in Figure S2) is computed. The process is repeated for ten times to compute the average Bhattacharyya distance of the distributions (See Equation 3 in the Main Text). The y-axis shows the difference between the average Bhattacharyya distance at each nRM and that at 10000 nRM. The minimum nRM is selected if the difference is below a threshold (0.0001, black line). The result of the minimum nRM for each circuit is shown in Fig. 3c.

**Figure S11. The dissimilarity of expressions of each gene for each coupled toggle-switch (CTS-I) circuit as a function of nRM.** nIC is fixed to 1000. Due to the symmetric of each circuit, only gene A_i_ for each circuit is plotted and colored differently for each gene.

**Figure S12.** **The minimum nRM required for the convergence of RACIPE using the distribution of gene expressions**. For each number of random models (nRM, different points) of each circuit (CTS-I, different panels), the distribution of the gene expression of each gene is computed. The process is repeated for ten times to compute the average Bhattacharyya distance of the distributions (See Equation 3 in the Main Text). The y-axis shows the difference between the average Bhattacharyya distance at each nRM and that at 10000 nRM. The minimum nRM is selected if the difference is below a threshold (0.001, black line). The result of the minimum nRM for each circuit is shown in Fig. 3d.

**Figure S13. Average linkage hierarchical clustering analysis of *in silico* simulated gene expression data for the cases of knockout treatments.** Each column corresponds to a gene, and each row corresponds to a stable steady state.

**
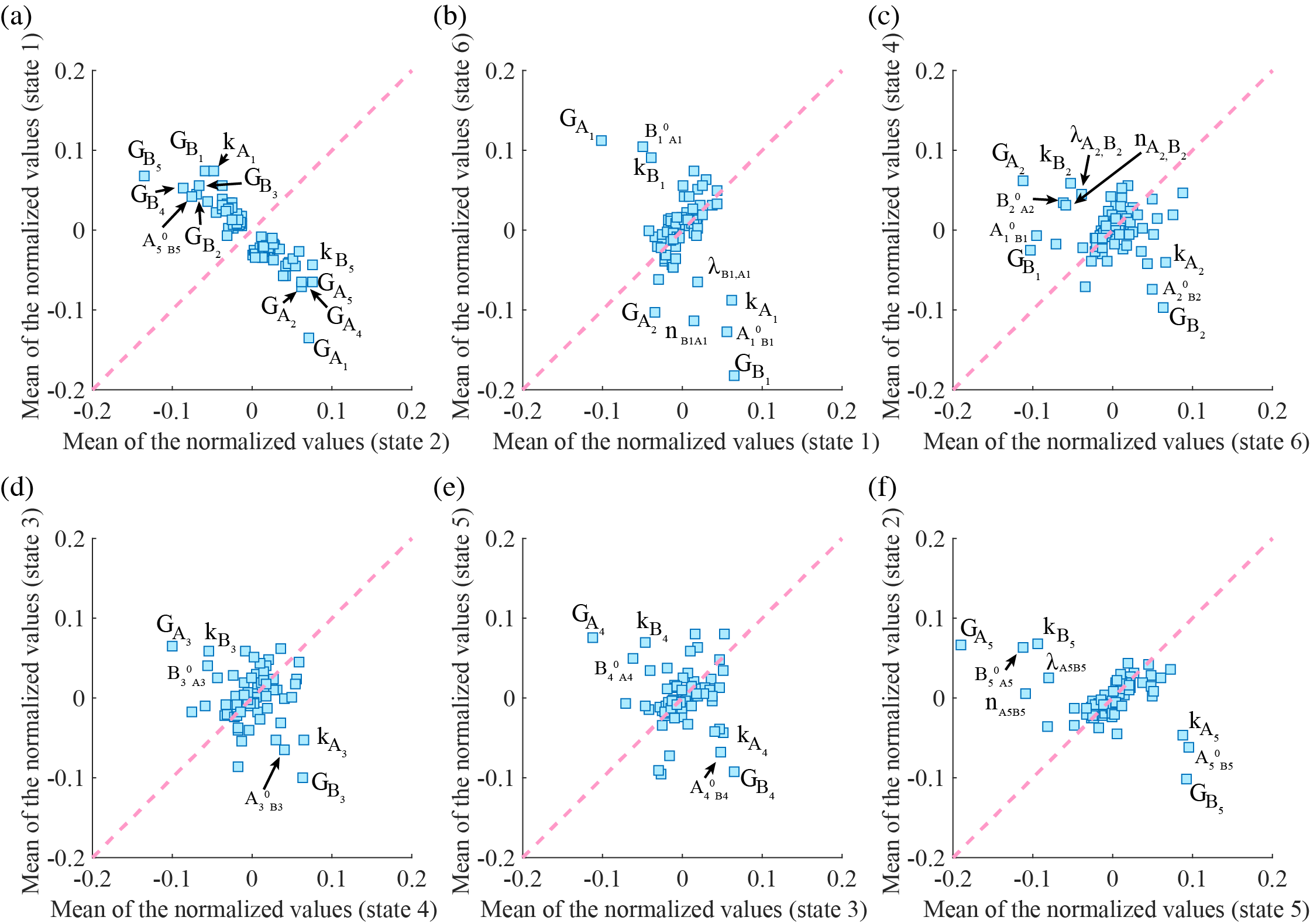
**

**Figure S14: Key parameters that are involved in the transitions among certain gene states.** (a) The mean of the normalized values for each parameter from all the models that have gene state 2 but not gene state 1 (x-axis) versus the mean calculated from all the models that have gene state 1 but not gene state 2 (y-axis). The most distinct parameters are highlighted in the plot. (b) gene states 1 and 6; (c) gene states 6 and 4; (d) gene states 4 and 3; (e) gene states 3 and 5; (f) gene states 5 and 2. $G_{x}$ and $k_{x}$ represent the production and degradation rate of gene x respectively, and $x_{y}^{0}$, $n_{xy}$ and $\lambda_{xy}$ represent the threshold, the Hill coefficient and the fold change for the regulation from gene $x$ to gene $y$.

**
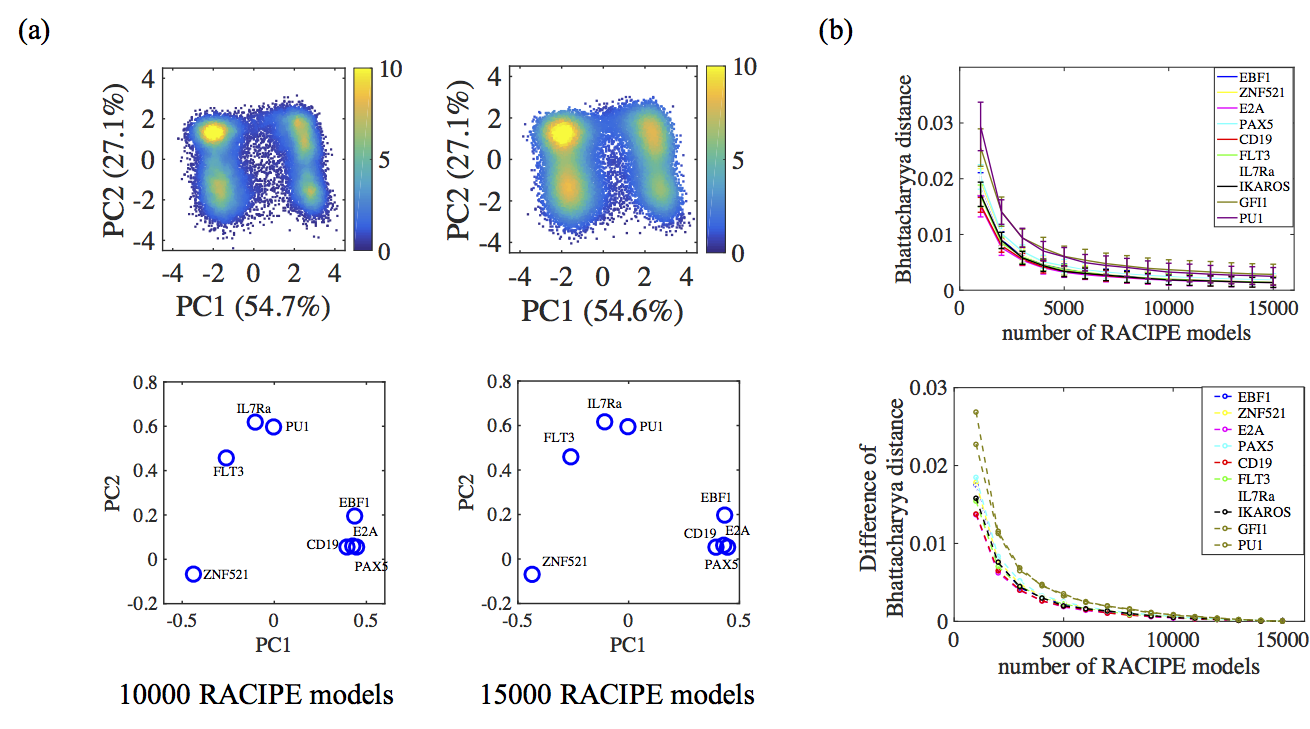
**

**Figure S15 Convergence test for the application of RACIPE on the gene circuit governing B-cell development (a)** Top panel: 2D probability density map of the RACIPE-predicted gene expression data projected to the first (PC1) and second principal component (PC2) axes, for 10000 and 15000 RACIPE models, respectively. Bottom panel: the PC1 and PC2 loadings of each gene for the corresponding density maps. (**b**) Top panel: The dissimilarity of expression distribution (See Equation 3 in the Main Text) of each gene among 5 repeats of RACIPE for the gene circuit governing B cell development as a function of nRM. nIC is fixed to 1000. Bottom panel: The difference between the average Bhattacharyya distance at each nRM and that at 15000 nRM. The increase of the number of models leads to the convergence of the divergences. Different colors represent different genes. Notably, since the TF IKAROS is purely an input and the TF GFI1 has only one upstream regulator IKAROS, the expression of IKAROS and GFI1 is not restricted by the topology of the network; thus we exclude both genes in the clustering analysis in Figure 6.

**4. Usage guide for RACIPE (also available as the Readme file)**

(1) Use "make" to compile and use "make clean" to clean up all the compiled files. The executable file is named as "RACIPE" by default.

(2) Run the package:

1> Run with the .topo file:

$ ./RACIPE *.topo [options]

RACIPE generates two files according to the topology information (in the .topo file):

- Configuration file (.cfg file) storing all the settings for the simulation (the .cfg file can be used in the second method to run the package, see below);

- Parameter file (.prs file) storing ranges of the parameters for randomization.

If the "-flag" option is set to 0 (default), RACIPE will calculate the results for all RACIPE models and generate "_parameter.dat", "_solution_.dat", and "_T_test.dat".

If the "-flag" option is set to 1, only the .cfg and .prs will be generated.

Example:

$make

$./RACIPE TS.topo [options]

2> Run with the .cfg file

$ ./RACIPE *.cfg [options]

.prs file will be automatically generated/updated according to the settings in .cfg file no matter the old .prs file exists or not.

To change the settings of the simulation, users can either change the .cfg file directly or use options. However, the options that are set in the command line will always override the settings in the .cfg file. New configuration file will be generated as "_tmp.cfg" in the same folder to store the simulation settings for the current run.

Similarily,

If the "-flag" option is set to 0 (default), RACIPE will calculate the results for all RACIPE models and generate "_parameter.dat", "_solution_.dat", and "_T_test.dat".

If the "-flag" option is set to 1, the updated configuration file will be generated as "_tmp.cfg" in the same folder.

(3) Options

Use "./RACIPE -h" to find all available options.

-h : Show all available options.

-maxtime : Maximum time for the simulation (Default 23.5 h).

-solver : The integrator method (1 --> Euler or 2 --> RK45) to solve the ODEs equations (Default 1).

-flag : run RACIPE to produce the .cfg file only or the whole simulation (Default 0, perform the whole simulation).

-KDID : Gene or link (See their ID in the .cfg file) to be knocked down.

-OEID : Gene (See their ID in the .cfg file) to be overexpressed. (follow by -OEFD)

-OEFD : Fold change to overexpress a gene (-OEID must be first set in the option, the value need to be bigger than 1). (Default 1) if the corresponding OEFD is not set, it will be set to 1.

-DEID : Gene (See their ID in the .cfg file) to be downexpressed. (follow by -DEFD)

-DEFD : Fold change to downexpress a gene (-DEID must be first set in the option, the value need to be bigger than 1). (Default 1) if the corresponding DEFD is not set, it will be set to 1.

Attention: The .prs file will be same as the one without Knockdown/downexpression/overexpression treatments. Multiple genes or links can be treated at the same time by putting multiple -KDID, -OEID, -DEID in the command line or modifying the .cfg file.

-dist : Distribution used for randomization:

1 ---> Uniform Distribution (Default);

2 ---> Gaussian Distribution;

3 ---> Exponential Distribution.

-SF : Scale the distribution ranges of all the parameters except for the hill coefficients, should be smaller than 1 (Default 1).

-num_findT : The number of simulations used to estimate the thresholds (Default 10000).

-num_paras : The number of RACIPE models to generate (Default 100).

-num_ode : The number of Random initial values to solve ODEs (Default 100).

-num_stability : The maximum number of stable states to save for one RACIPE model (Default 10).

-thrd : Cutoff for convergence of steady states for numerically solving ODEs (Default 1.0).

-Toggle_f_p : Save parameters of each RACIPE model or not (Default 1 (yes)).

-stepsize : Stepsize for solving ODEs (Default 0.1).

-maxiters : The maximum number of iterations for solving ODEs at each random initial condition (Default 20).

-Toggle_T_test : Test the half-function rule (Default 1 (yes)).

-SBML_model : Output a model in the SBML format. The parameter will be the ID of the model (start from 1) to save (Default 0 (no SBML output)).

-seed : random seed (Default 1).

Attention: the actual seed used by the package is a function of the starting time and the seed set here. -seed is used for the case you run the package at the same time for the same circuit several times.

-minP : Minimum production rate (Default 1.0).

-maxP : Maximum production rate (Default 100.0).

-minK : Minimum degradation rate (Default 0.1).

-maxK : Maximum degradation rate (Default 1.0).

-minN : Minimum Hill coefficient (Default 1.0).

-maxN : Maximum Hill coefficient (Default 6.0).

-minF : Minimum fold change (Default 1.0).

-maxF : Maximum fold change (Default 100.0).

(4) Input files

1> Topology file (.topo file)

Format of the topology file, such as TS.topo:

Source Target Type

A B 1

... ... ...

Types of regualtions: 1 --> Activation; 2 --> Inhibition;

Or

2> Configure file (.cfg file), generated by the package. See details below.

(5) Output files

1> Standard output on screen, which can be redirected to the other file.

It contains the topology information, the result of testing the half-functional rule for each gene, the information of system stability, and running time.

2> configure file (.cfg file).

It generated by the package, contains the settings for the simulation, the ID for each gene (two columns: ID, Gene name) and regulatory links (four columns: ID, Gene ID, Gene ID, Type of regulation (consistent with the topology file))

Attention:

1. When directly modifying the .cfg file to use different random distributions, users should change the number after 'Distribution', and replace the name of the distribution after the number (optional).

2. When directly modifying the .cfg file for knockdown genes or links, users should change the 'number_of_KDs' to be the total number of genes and links to be knocked down, and put the Genes' ID and Links' ID after 'KD_ID' separated by space or tab.

3. When directly modifying the .cfg file for over/down expression of a gene, users should change the 'number_of_OEs' and/or 'number_of_DEs' to the genes to be treated, and put the gene IDs after 'OE_ID' and/or 'DE_ID'; users also need to set up the fold changes after 'OE_Fold_Change' and/or 'DE_Fold_Change'.

3> Parameter file (.prs file).

It is generated by the package, and it contains ranges of the parameters for randomization. It would be updated when users run with the .cfg file.

4> Temporary configure file (_tmp.cfg), generated when the package runs with the .cfg file; it contains the updated settings for the simulation.

5> _parameter.dat which contains the parameters of each RACIPE model.

Format of _parameter.dat:

Model_index Number_of_stable_states Parameters_of_model

number_of_stable_states : The number of stable steady states of the RACIPE model.

Parameters_of_model : The meaning of each column is in the same order as the parameters in the .prs file.

6> _solution_.dat which contains the gene expression for each stable steady state in log2 scale.

The models with different number of stable states are stored in different files, e.g. monostable models are stored in _solution_1.dat, and bistable models are stored in _solution_2.dat.

Format of _solution_.dat:

Model_index Number_of_stable_states Solutions

Solutions : The meaning of each column is consistent with the order of gene IDs in the .cfg file.

7> _T_test.dat storing the test of the half functional rule for each RACIPE model.

Format of _T_test.dat:

Model_index Over_threshold_A Below_threshold_A ...

Over_threshold_A : The number of stable states for the current RACIPE model whose expression of gene A is larger than its threshold parameter of A.

Below_threshold_A : The number of stable states for the current RACIPE model whose expression of gene A is smaller than its threshold parameter of A.

For each model, the probability for gene A's expression to be larger than its threshold equals to the sum of Over_threshold_A across all the models divided by the sum of both Over_threshold_A and Below_threshold_A across all the models.

**Reference:**

de Hoon,M.J.L. *et al.* (2004) Open source clustering software. *Bioinforma. Oxf. Engl.*, **20**, 1453–1454.

Huang,B. *et al.* (2017) Interrogating the topological robustness of gene regulatory circuits by randomization. *PLOS Comput. Biol.*, **13**, e1005456.

Saldanha,A.J. (2004) Java Treeview—extensible visualization of microarray data. *Bioinformatics*, **20**, 3246–3248.

van Zelm,M.C. *et al.* (2005) Ig gene rearrangement steps are initiated in early human precursor B cell subsets and correlate with specific transcription factor expression. *J. Immunol. Baltim. Md 1950*, **175**, 5912–5922.
